# Supplementary material for: Active transcriptome remodeling underlies antimony-induced drug tolerance in persister-like Leishmania parasites
Source: PLoS Pathog. 2026 Oct 5;22(10):e1014682. doi: 10.1371/journal.ppat.1014682 (PMC13649148; doi:10.1371/journal.ppat.1014682)
Supplement: Supplementary file 1 [file ppat.1014682.s001.pdf]

S1 Fig.

A

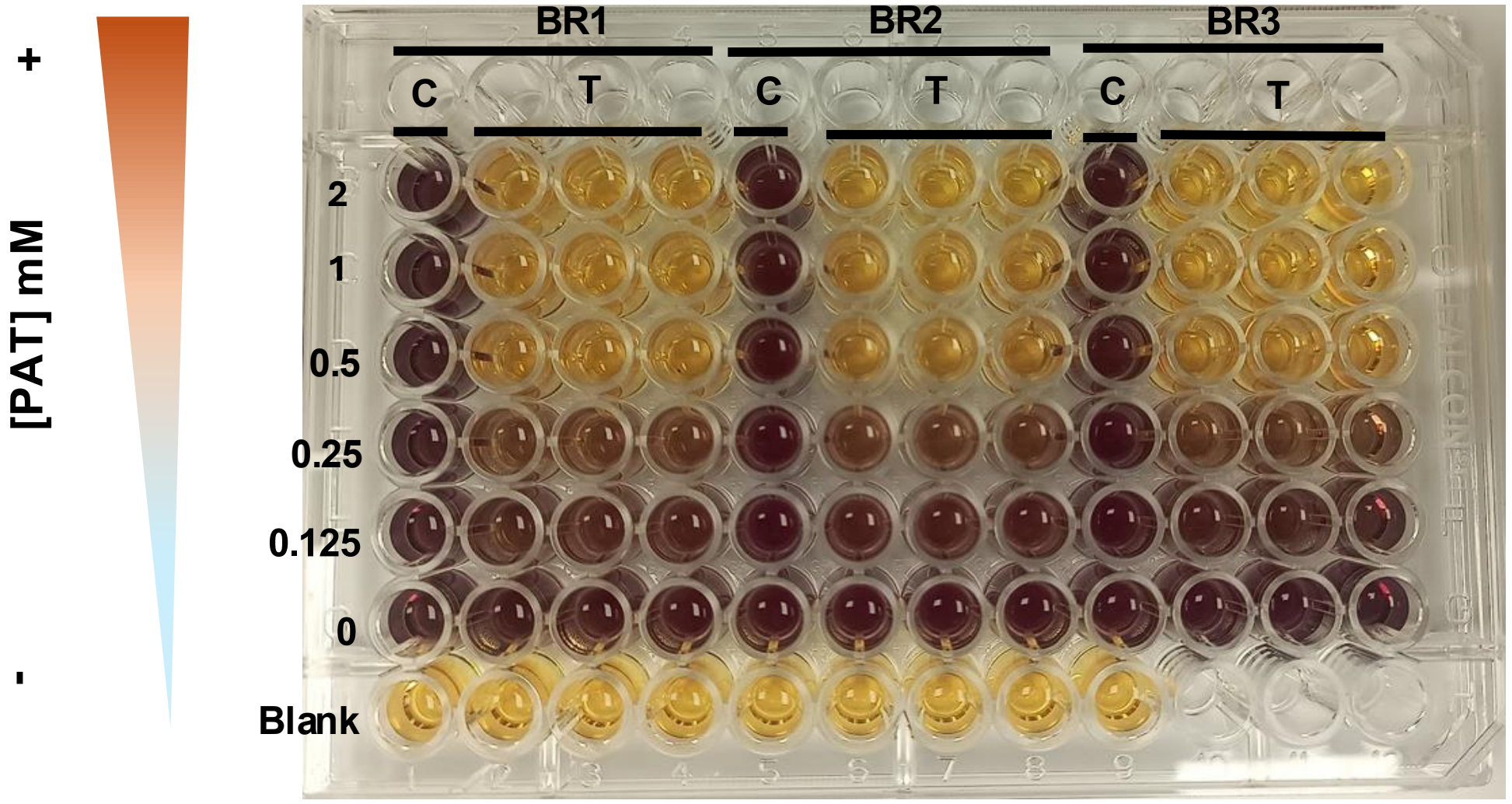
+
[PAT] mM
-
BR1
BR2
BR3
C
T
C
T
C
T
2
1
0.5
0.25
0.125
0
Blank

B

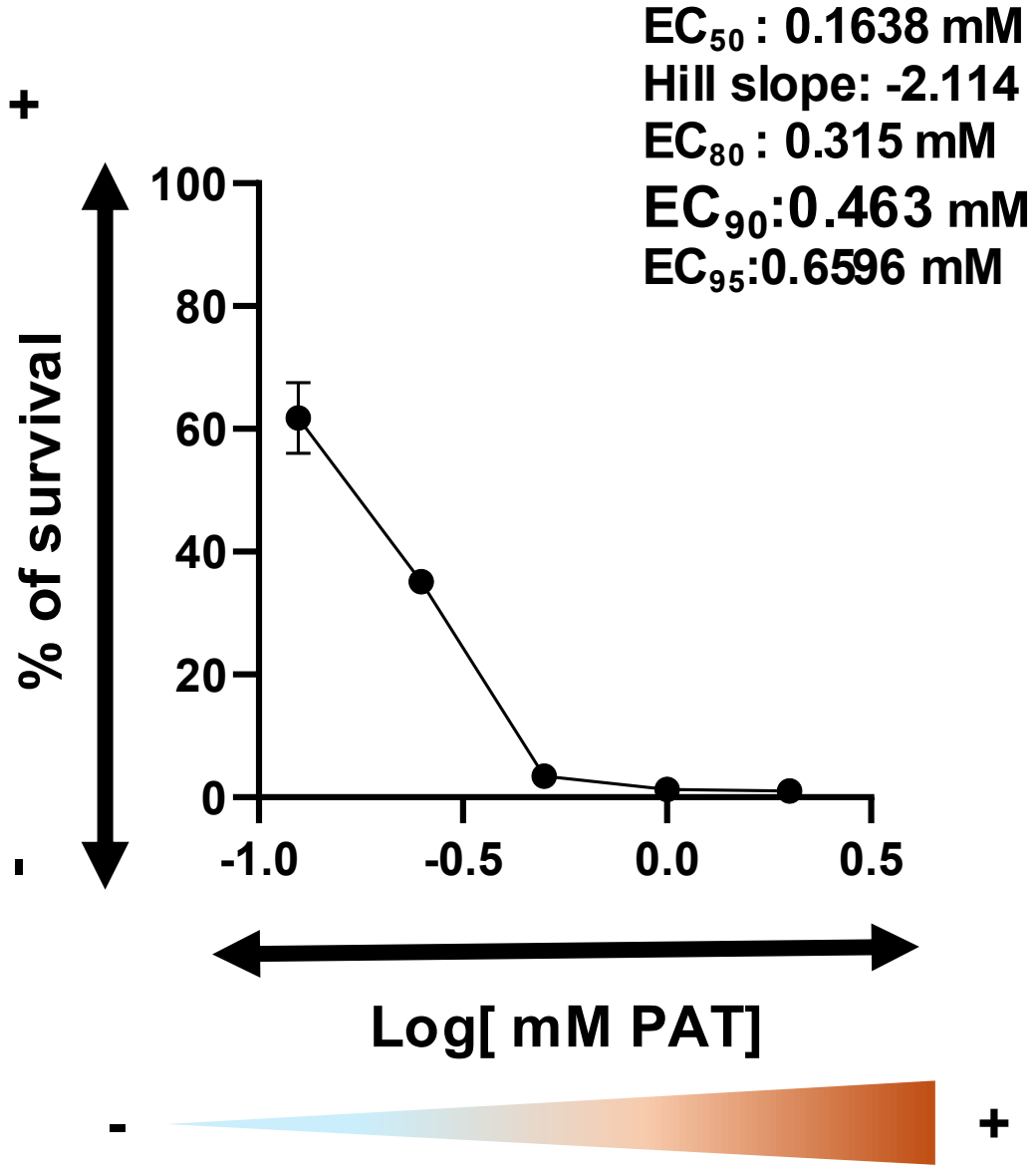
EC50 : 0.1638 mM
Hill slope: -2.114
EC80 : 0.315 mM
EC90:0.463 mM
EC95:0.6596 mM
+
-
% of survival
100
80
60
40
20
0
-1.0
-0.5
0.0
0.5
Log[mM PAT]
-
+
