# Supplementary material for: Active transcriptome remodeling underlies antimony-induced drug tolerance in persister-like Leishmania parasites
Source: PLoS Pathog. 2026 Oct 5;22(10):e1014682. doi: 10.1371/journal.ppat.1014682 (PMC13649148; doi:10.1371/journal.ppat.1014682)

S2 Fig.

A

Parental (No drug challenge)

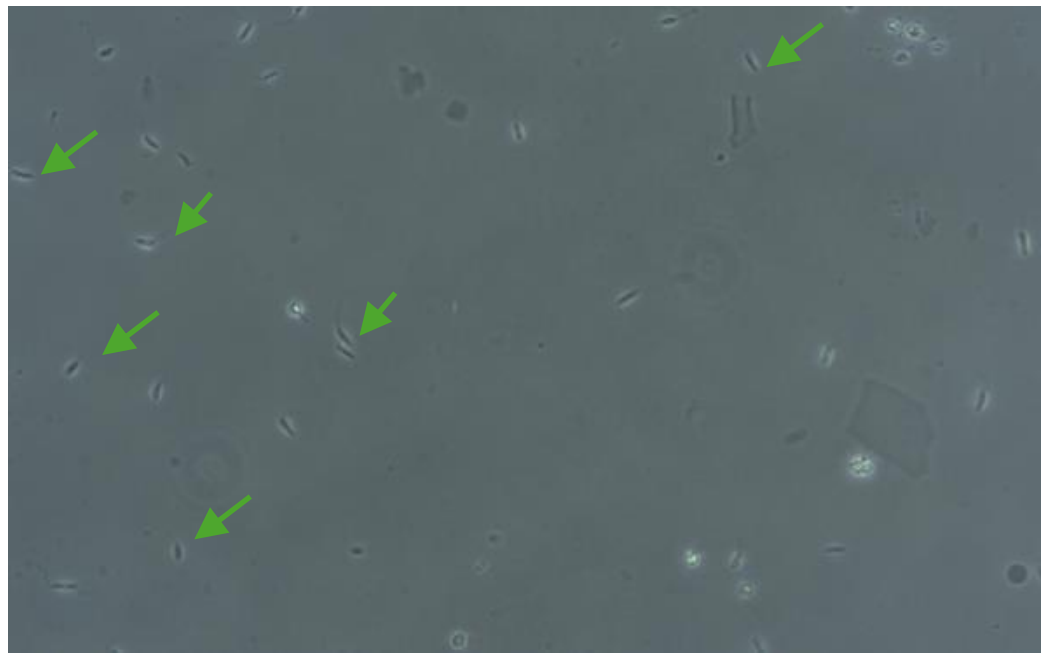

B

Persister-like (after drug challenge)

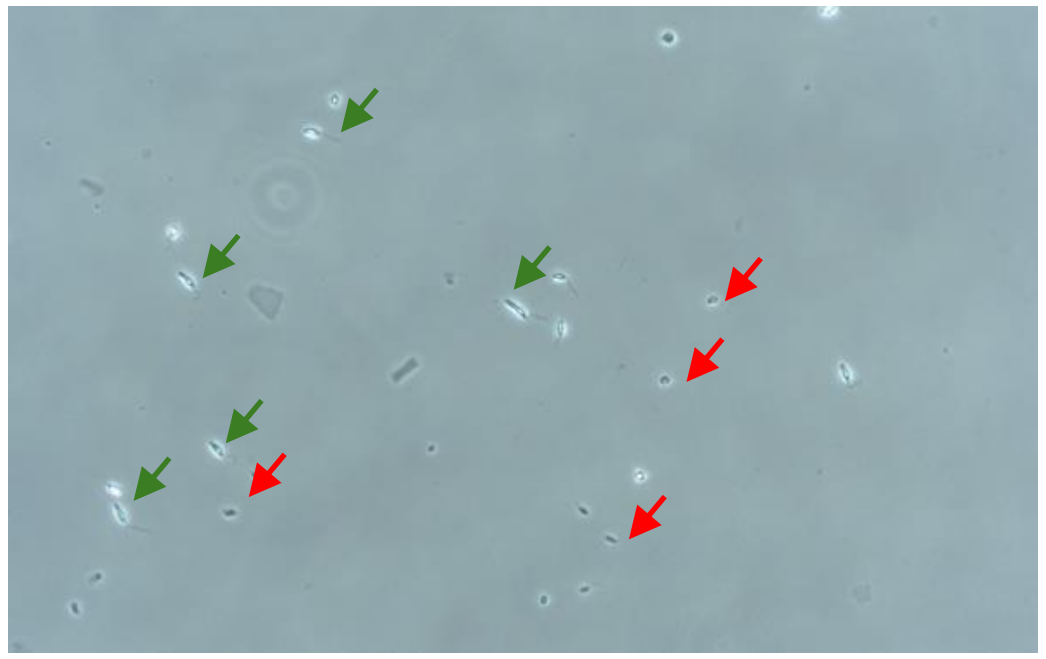

Supplement: Supplementary file 2 [file ppat.1014682.s002.pdf]
