# Supplementary material for: Active transcriptome remodeling underlies antimony-induced drug tolerance in persister-like Leishmania parasites
Source: PLoS Pathog. 2026 Oct 5;22(10):e1014682. doi: 10.1371/journal.ppat.1014682 (PMC13649148; doi:10.1371/journal.ppat.1014682)
Supplement: Supplementary file 3 [file ppat.1014682.s003.pdf]

S3 Fig.

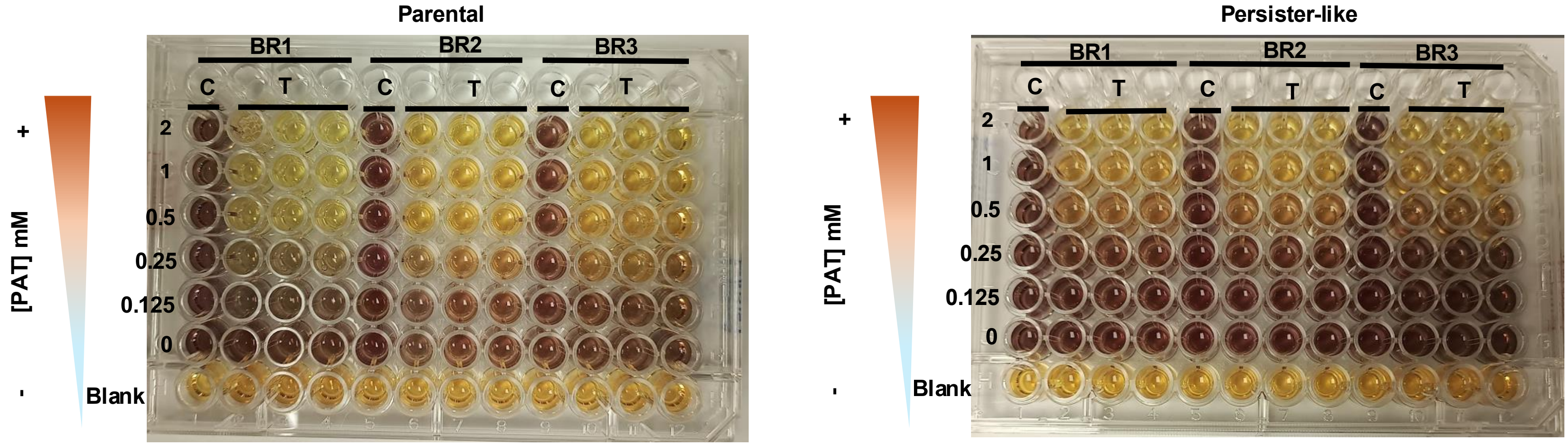

Parental
EC_{50} : **0.1454** mM
Hill slope: -2.373
$LogEC_{50}$:-0.8375

Persisters-like
EC_{50} : **0.3434** mM
Hill slope: -2,523
$LogEC_{50}$: -0,4643
