# Supplementary material for: Active transcriptome remodeling underlies antimony-induced drug tolerance in persister-like Leishmania parasites
Source: PLoS Pathog. 2026 Oct 5;22(10):e1014682. doi: 10.1371/journal.ppat.1014682 (PMC13649148; doi:10.1371/journal.ppat.1014682)
Supplement: Supplementary file 4 [file ppat.1014682.s004.pdf]

S4 Fig.

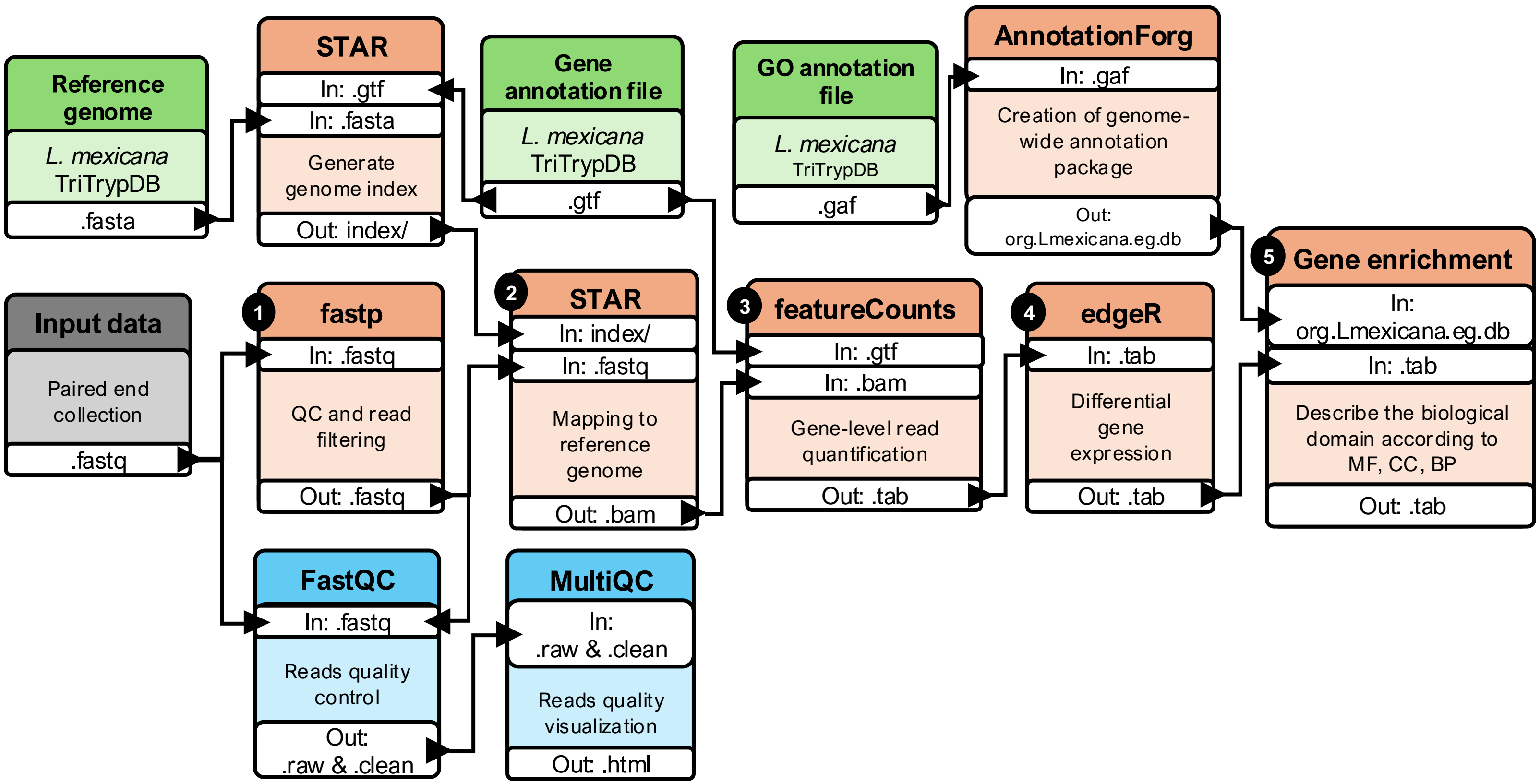
Reference genome
L. mexicana TriTrypDB
.fasta
STAR
In: .gtf
In: .fasta
Generate genome index
Out: index/
Gene annotation file
L. mexicana TriTrypDB
.gtf
GO annotation file
L. mexicana TriTrypDB
.gaf
AnnotationForg
In: .gaf
Creation of genome-wide annotation package
Out: org.Lmexicana.eg.db
5 Gene enrichment
In: org.Lmexicana.eg.db
In: .tab
Describe the biological domain according to MF, CC, BP
Out: .tab
Input data
Paired end collection
.fastq
1 fastp
In: .fastq
QC and read filtering
Out: .fastq
2 STAR
In: index/
In: .fastq
Mapping to reference genome
Out: .bam
3 featureCounts
In: .gtf
In: .bam
Gene-level read quantification
Out: .tab
4 edgeR
In: .tab
Differential gene expression
Out: .tab
FastQC
In: .fastq
Reads quality control
Out: .raw & .clean
MultiQC
In: .raw & .clean
Reads quality visualization
Out: .html
