# Supplementary material for: Active transcriptome remodeling underlies antimony-induced drug tolerance in persister-like Leishmania parasites
Source: PLoS Pathog. 2026 Oct 5;22(10):e1014682. doi: 10.1371/journal.ppat.1014682 (PMC13649148; doi:10.1371/journal.ppat.1014682)
Supplement: Supplementary file 5 [file ppat.1014682.s009.docx]

**Reviewers' Comments:**

Reviewer's Responses to Questions

**Part I - Summary**
Please use this section to discuss strengths/weaknesses of study, novelty/significance, general execution and scholarship.

*Reviewer #1: The manuscript by Quienco et al., describes an experimental system using an in vitro drug exposure/recovery/rechallenge model in promastigotes. The terminology used throughout the manuscript is problematic. The authors refer to drug-surviving axenic promastigotes as “persisters” and repeatedly link this model to persistence and clinical relapse. However, persistent Leishmania infection is generally studied in the context of host infection, particularly intracellular amastigotes within mammalian cells or tissues, rather than axenic promastigote cultures. The phenotype observed in vitro in their drug-exposure/recovery/rechallenge model i.e., survival after EC80 antimony exposure, delayed regrowth, and increased EC50 upon rechallenge, is more accurately described as drug tolerance, stress adaptation, or drug-tolerant promastigote survival. Unless the authors demonstrate reversibility, lack of heritable resistance, absence of genomic amplification/mutation, and relevance in intracellular amastigotes or an infection model, the term “persister” should be used with substantial caution, preferably as “persister-like,” and the title, abstract, and conclusions should be revised to avoid conflating this phenotype with persistent Leishmania infection.*

We thank the reviewer for their insightful, constructive, and rigorous evaluation of our manuscript. Please see our responses to each comment below.

*1) The term "persistence" as commonly understood in the context of infectious disease carries specific connotations that the axenic promastigote model does not fully satisfy. In in vivo settings, persistence occurs within phagocytic cells (macrophages), where parasites must overcome host immune mechanisms including reactive oxygen and nitrogen species, acidic pH, nutrient limitation, and active immune surveillance. None of these pressures are recapitulated in axenic promastigote culture employed in this study. The drug pressure applied here is a single, defined chemical stressor, a significant simplification of the hostile host environment.*

Response:

We fully agree that the term "persisters" in Leishmania research is most appropriately evaluated within host infection models (using intracellular amastigotes). However, establishing a robust persistence model *in vivo* remains exceptionally challenging and represents a major goal for our future work. Our current study is among the first to investigate the potential mechanisms governing increased drug tolerance and persister-like phenotypes in Leishmania promastigotes. Understanding molecular mechanisms of persisters is still at infancy due to the lack of suitable in vivo models and difficulties to isolate persisters. Nevertheless, we fully recognize this limitation. In accordance with the reviewer's excellent recommendations, we have thoroughly revised the manuscript. We have pivoted our terminology away from definitive "persisters" to **"drug-tolerant promastigotes"** or **"persister-like cells" and modified and revised our conclusions accordingly. Moreover, we have incorporated** the reviewer’s exact points into our new **Study Limitations and Future Directions section at the end of Discussion, please see pages 16-17.**

*2) Amastigote persistence within macrophages involves complex host-parasite interactions, including manipulation of host cell signaling, evasion of phagolysosomal killing, and modulation of antigen presentation. Promastigotes in axenic culture exist in none of this biological context.*

Response:

We agree that the axenic promastigote model is a simplified system. It lacks the complex conditions found in vivo, such as host cell signaling, evasion of phagolysosomal killing, and antigen modulation. We also recognize that testing drug tolerance under a single chemical stressor does not fully capture how infections persist in a living host. However, the goal of this study was to isolate the intrinsic, cell-autonomous traits of drug-tolerant parasites without host cell interference. Furthermore, bacterial and cancer research shows that in vitro models successfully reveal core persistence strategies. Establishing a robust in vivo persistence model in *Leishmania* remains a major challenge that we discussed in our newly added Study limitations and Future directions section. In our future directions we plan to establish host intracellular amastigote models and in vivo systems, however, it would require substantial optimization efforts and currently is not feasible. Please see pages 16-17.

*3) The promastigote parasites are adapted to the sandfly midgut environment, not the mammalian host. Their baseline biology including their metabolic preferences, surface molecule expression, and stress response repertoire differs substantially from amastigotes. Drug responses observed in promastigotes may not translate directly to amastigote behavior in vivo.*

Response:

We fully agree with the reviewer’s accurate assessment regarding the biological divergence between the promastigote and amastigote stages of Leishmania. Our use of promastigotes in this study was intended to establish a model where parasites exhibit increased persister-like properties and perform initial investigation of pathways that underlie increase in the drug tolerance. In Leishmania, both promastigotes and amastigotes share fundamental biochemical responses to active trivalent antimony including the disruption of thiol redox metabolism via trypanothione reductase inhibition, drug influx through aquaglyceroporins, and activation of ATP-binding cassette (ABC) efflux pathways during resistance. The active trivalent antimony enters the cells of both promastigotes and amastigotes using similar membrane machinery, primarily through the aquaglyceroporin channel **AQP1 [1].** Resistant strains in both developmental stages rely on ATP-binding cassette transporters to bind antimony-thiol conjugates and pump them out of the cytosol or sequester them into internal vesicles [2]. Therefore, we anticipate overlapping drug responses in both promastigotes and amastigotes.

1. Frézard F, Monte-Neto R, Reis PG. Antimony transport mechanisms in resistant leishmania parasites. Biophys Rev. 2014 Mar;6(1):119-132. doi: 10.1007/s12551-013-0134-y. Epub 2014 Jan 25. PMID: 28509965; PMCID: PMC5425714.
2. Douanne N, Wagner V, Roy G, Leprohon P, Ouellette M, Fernandez-Prada C. MRPA-independent mechanisms of antimony resistance in Leishmania infantum. Int J Parasitol Drugs Drug Resist. 2020 Aug; 13:28-37. doi: 10.1016/j.ijpddr.2020.03.003. Epub 2020 Apr 2. PMID: 32413766; PMCID: PMC7225602.

*4) The EC80 PAT challenge used in this study selects for cells that can survive a specific chemical insult. Whether the surviving cells are genuinely "persisters" in the biological sense, or simply the tail of a normal distribution of drug sensitivity within the population, is not definitively resolved by the data presented. The authors do not perform single-cell analyses or lineage tracing to confirm that the surviving cells are a pre-existing subpopulation rather than stochastically drug-tolerant cells.*

Response:

We thank the reviewer for the opportunity to clarify our methodology. Subjecting microorganisms or cancer cells to high drug concentrations is a standard approach to generate persister-like cells and investigate mechanisms driving increased drug tolerance and persister-like features using bulk RNA-seq and numerous papers have been published using this approach [1-6]. We have added a clarification sentence in the Introduction stating that exposure to drug is a commonly used approach to enrich for cells with persister-like features and introduced corresponding references [1-6], please see lines 68-72 in unmarked clean version.

1. Keren I, Shah D, Spoering A, Kaldalu N, Lewis K. Specialized persister cells and the mechanism of multidrug tolerance in Escherichia coli. J Bacteriol. 2004 Dec;186(24):8172-80. doi: 10.1128/JB.186.24.8172-8180.2004. PMID: 15576765; PMCID: PMC532439.
2. Keren I, Minami S, Rubin E, Lewis K. Characterization and transcriptome analysis of Mycobacterium tuberculosis persisters. mBio. 2011 Jun 14;2(3):e00100-11. doi: 10.1128/mBio.00100-11. PMID: 21673191; PMCID: PMC3119538.
3. Oren Y. Standing on the shoulders of microbes: How cancer biologists are expanding their view of hard-to-kill persister cells. Mol Syst Biol. 2022 Jul;18(7):e11168. doi: 10.15252/msb.202211168. PMID: 35866381; PMCID: PMC9305393.
4. Shen, S., Faouzi, S., Bastide, A. *et al.* An epitranscriptomic mechanism underlies selective mRNA translation remodelling in melanoma persister cells. Nat Commun 10, 5713 (2019). https://doi.org/10.1038/s41467-019-13360-6.
5. Windels, E.M., Ben Meriem, Z., Zahir, T. *et al.* Enrichment of persisters enabled by a ß-lactam-induced filamentation method reveals their stochastic single-cell awakening. *Commun Biol* **2**, 426 (2019). https://doi.org/10.1038/s42003-019-0672-3.
6. de Oliveira BCD, Arias Del Angel J, Herrmann May N, Barrett MP, Domagalska MA, Beneke T. Beyond resistance: Emerging methods to dissect drug responses in Leishmania. PLoS Pathog. 2026 Jun 24;22(6):e1014331. doi: 10.1371/journal.ppat.1014331. PMID: 42340977; PMCID: PMC13293385.

*5) Leishmania parasites use polycistronic transcription, which is an important context here. Trypanosomatids largely lack conventional single-gene transcriptional regulation; genes are transcribed in polycistronic transcription units and mature mRNAs are generated by trans-splicing and polyadenylation, with much regulation occurring post-transcriptionally. The claims about 'active transcriptome remodeling' is not impossible, but it needs to be interpreted carefully. RNA abundance changes in Leishmania can reflect mRNA stability, processing, copy-number variation, translation-linked RNA turnover, aneuploidy, or selection of subpopulations, not necessarily classical transcriptional activation/repression.*

Response:

We thank the reviewer for highlighting this critical context regarding Leishmania biology. Yes, trypanosomatids rely on polycistronic transcription and that classical single-gene transcriptional regulation is absent. Our intended meaning by "active transcriptome remodeling" was to describe the significant shifts in steady-state mRNA abundance, rather than classical promoter-driven transcriptional activation or repression. To address this and prevent any misunderstanding we clarified it in the Results section explicitly stating that these observed changes likely reflect post-transcriptional mechanisms (such as altered mRNA stability, trans-splicing efficiency, or translation-linked RNA turnover etc) and added the relevant reference, please see lines 229-223 in unmarked clean version.

*6) Following on the comment above, the claims regarding “epigenetic adaptations” are not sufficiently supported by the presented data. Leishmania does possess chromatin-associated regulatory mechanisms, including histone modifications and base J, but gene expression in this parasite is organized primarily through polycistronic transcription units and extensive post-transcriptional regulation. Therefore, changes in steady-state mRNA abundance cannot be straightforwardly interpreted as evidence of epigenetic regulation. The manuscript appears to infer epigenetic remodeling mainly from differential expression of putative histone-modifying enzymes. This is insufficient to support the claim made in the title of the manuscript that epigenetic adaptations underlie the phenotype. Direct evidence, such as ChIP-seq/ChIP-qPCR for relevant histone marks, base J mapping, ATAC-seq/MNase-seq, or perturbation of the proposed epigenetic regulators, would be required. In the absence of such data, the authors should substantially tone down the epigenetic claims and describe them as hypotheses generated from transcriptomic data. The authors are clearly overinterpreting the data.*

Response:

We agree that while our data support the involvement of epigenetic control, specific claims regarding epigenetic adaptations remain premature. Therefore, we removed 'epigenetic adaptations' from the title, softened our conclusions, and reframed these ideas as hypotheses derived from our transcriptomic data, lines 493-495 in unmarked clean version.

A new title: Active Transcriptome Remodeling Underlies Antimony-Induced Drug Tolerance in persister-like Leishmania Parasites
 *7) Overall, the manuscript is presented as a study of Leishmania persistence, but the data primarily support an in vitro model of antimony-induced drug tolerance/adaptation in axenic promastigotes. The title, abstract, and discussion overstate the connection to persistent infection and clinical relapse. The terminology is especially confusing because persistent Leishmania infection is usually associated with intracellular amastigote stages in host cells or tissues, whereas this study uses promastigote cultures. In addition, the claims of “epigenetic adaptations” are not directly demonstrated. Given the polycistronic organization of Leishmania gene expression and the dominant role of post-transcriptional regulation, RNA-seq changes alone do not establish epigenetic remodeling. The manuscript would be stronger and more accurate if reframed as a study of drug-tolerant promastigote survival and transcriptomic/post-transcriptional adaptation following antimony exposure, with epigenetic mechanisms discussed only as a speculative possibility requiring direct validation.*

Understanding the molecular mechanisms of persisters remains in its infancy. This is due to a lack of suitable in vivo models and the difficulty of isolating these cells. Currently, generating persister-like cells using high doses of antibiotics is a standard procedure in bacteria and a similar approach is used in cancer cells [1-3]. As a recent paper on bacterial persisters notes: "It can be seen that how to distinguish between tolerance and persistence has remained somewhat ambiguous so far, and no one has been able to pinpoint their exact difference. We hold the opinion that there might be no fundamental difference between persisters and 'tolerant bacteria'..." [4]. According to the reviewer request, **we have nuanced our language to persister-like across the manuscript** and added a clarifying statement to the Introduction. This addition specifies that **drug exposure is a standard method** used to enrich for cells with persister-like features across various organisms, and we have included the corresponding references to support this point [1-4], please see lines 68-72 in unmarked clean version.

Our current study is among the first to investigate the potential mechanisms governing increased drug tolerance and persister-like phenotypes in Leishmania parasites. The primary goal was to isolate the intrinsic, cell-autonomous traits of drug-tolerant parasites without host cell interference. Establishing an intracellular amastigote persister model requires substantial time and is reserved for our future directions. We find promastigote model very useful because both promastigotes and amastigotes share fundamental biochemical responses to active trivalent antimony including the disruption of thiol redox metabolism via trypanothione reductase inhibition, drug influx through aquaglyceroporins, and activation of ATP-binding cassette (ABC) efflux pathways, as shown in Table 2.

In accordance with the reviewer's recommendations, we have thoroughly revised the manuscript. We have pivoted our terminology away from definitive "persisters" to **"drug-tolerant promastigotes"** or **"persister-like cells" and modified and revised our conclusions accordingly. Regarding the epigenetic mechanisms as mentioned above we removed epigenetic adaptations from the title,** toned down the epigenetic claims in the manuscript and described them as hypotheses based on our transcriptomic data, lines 493-495 in unmarked clean version.

References associated with this comment:

1. Cui P, Niu H, Shi W, Zhang S, Zhang W, Zhang Y. Identification of Genes Involved in Bacteriostatic Antibiotic-Induced Persister Formation. Front Microbiol. 2018 Mar 6;9:413. doi: 10.3389/fmicb.2018.00413. PMID: 29559967; PMCID: PMC5845583.

2. Li Y, Zhang L, Zhou Y, Zhang Z, Zhang X. Survival of bactericidal antibiotic treatment by tolerant persister cells of Klebsiella pneumoniae. J Med Microbiol. 2018 Mar;67(3):273-281. doi: 10.1099/jmm.0.000680. Epub 2018 Jan 19. PMID: 29458540.

3. Shen, S., Faouzi, S., Bastide, A. *et al.* An epitranscriptomic mechanism underlies selective mRNA translation remodelling in melanoma persister cells. *Nat Commun* **10**, 5713 (2019). https://doi.org/10.1038/s41467-019-13360-6.

4. Niu, H., Gu, J. & Zhang, Y. Bacterial persisters: molecular mechanisms and therapeutic development. *Sig Transduct Target Ther* **9**, 174 (2024). https://doi.org/10.1038/s41392-024-01866-5.

*ZK Reviewer #2: This manuscript presents a well-designed and comprehensive study addressing a significant gap in our understanding of treatment failure in leishmaniasis. The authors establish an in vitro model using Ficoll gradient centrifugation to isolate persister-like Leishmania mexicana parasites and utilize RNA-seq to investigate the molecular mechanisms of persistence. A major strength of the study is the multi-stage experimental design (induction, recovery, and re-challenge), which reveals that persistence is an active, orchestrated process rather than a passive metabolic shutdown. Furthermore, the identification of a "transcriptomic memory" in re-challenged persisters is a highly valuable contribution to the field. While the data is robust and the overall conclusions are sound, the manuscript would benefit from adjustments to its narrative, improved data visualization, and expanded discussion of the model's limitations.*

We thank the reviewer for their insightful, constructive, and rigorous evaluation of our manuscript and for recognizing the impact of this study. Please see our responses to each comment below under Part II.

*Reviewer #3: In the manuscript entitled “Active transcriptome remodeling and epigenetic adaptations underlie persistence in Leishmania parasites” Quiceno and colleagues perform transcriptome analysis in a model of parasite persistence. They have demonstrated the biological processes and pathways involved in the generating of persistence after drug treatment and the changes once the treatment is removed. I consider this paper to be relevant for improving drug treatments against leishmaniasis. Still, some questions and comments should be addressed.*

We thank the reviewer for their insightful, constructive, and rigorous evaluation of our manuscript and for recognizing the impact of this study. Please see our responses to each comment below under Part II.

**Part II – Major Issues: Key Experiments Required for Acceptance**
Please use this section to detail the key new experiments or modifications of existing experiments that should be absolutely required to validate study conclusions.

Generally, there should be no more than 3 such required experiments or major modifications for a "Major Revision" recommendation. If more than 3 experiments are necessary to validate the study conclusions, then you are encouraged to recommend "Reject".

*Reviewer #1: 1) The metabolic restriction in amastigotes reported in studies of parasite persistence by Beverley et al., and McConville et al., report it as a developmental program, not a stress response. The slow doubling time and restricted metabolism of amastigotes reported by Beverley et al., reflect a genetically and epigenetically encoded developmental differentiation, not a transient phenotypic adaptation to drug pressure. Conflating the two risks overstating the novelty of the persister phenotype described in this study, since some of what is being observed in drug-stressed promastigotes may simply reflect partial activation of the amastigote developmental program under stress — a well-known phenomenon in Leishmania biology.*

Response:

We thank the reviewer for this critical distinction. We agree that distinguishing between a drug-induced stress response and the pre-programmed amastigote developmental differentiation is vital for accurate interpretation. While we acknowledge that some metabolic restrictions may overlap with the amastigote program, we believe there are distinct differences in how Leishmania responds specifically to trivalent antimony compared to normal developmental cues (like pH and temperature shifts). The tolerance phenotype we observe involves the specific modulation of antimony-handling machinery. This includes the downregulation of aquaglyceroporins (drug influx) and the activation of ATP-binding cassette (ABC) efflux pathways (Table 2). These are specific mechanisms to survive chemical toxicity, rather than standard components of the developmental differentiation program described by Beverley et al. and McConville et al. We have expanded the Discussion section to explicitly contrast the antimony-specific stress response from the general amastigote differentiation program and added related references (Beverley et al. and McConville et al.,), please see a new paragraph, lines 534-543 in unmarked clean version in the Discussion section.

*Reviewer #2: 1. Presentation and Visualization of Transcriptomic Data
While the text provides data supporting the claim of "extensive transcriptome remodeling," the current presentation relies heavily on dense textual descriptions of gene counts. This makes it difficult for the reader to understand the scale, direction, and dynamics of these drastic changes.

Please add a consolidated table or a stacked bar chart clearly summarizing the absolute number of up-regulated versus down-regulated genes across all comparisons side-by-side. Consolidate the Results text.*

Response:

Thank you for this helpful suggestion. We have added a new **summary table** (now Table 1) that compares the **up-regulated and down-regulated genes** side-by-side for all conditions and consolidated text in the Results, page 11.

*2. Premature Conclusion of a "Dormant State"
The conclusion that persister parasites enter a "dormant state" is introduced prematurely in the first Results section. The primary evidence presented at that stage is a population-level growth curve (Fig. 2A) demonstrating delayed proliferation. While consistent with dormancy, this correlational phenotype could equally stem from a generalized recovery from cellular damage.

Please present the findings strictly as phenotypes (delayed proliferation and enhanced drug tolerance). Reserve the mechanistic claim of a "dormant state" until after presenting the transcriptomic data, which provides the necessary molecular evidence (e.g., global downregulation of biosynthetic processes) to support regulated quiescence.*

Response:

Thank you for this insightful comment. We agree that concluding a "dormant state" based solely on Fig. 2A is premature, as delayed proliferation can also reflect general recovery from cellular damage. Following your guidance, we have revised the first Results section to describe the findings strictly in terms of phenotypes (**delayed proliferation** and **enhanced drug tolerance, please see lines 215-218** in unmarked clean version). We have reserved the mechanistic claim of a "dormant state" for the later section of the manuscript, where it is properly contextualized and supported by our transcriptomic data demonstrating the **global downregulation of biosynthetic processes**.

*3. Clarifying the Concept and Evidence for "Molecular Memory"
The authors repeatedly refer to "molecular memory," yet this central concept is neither explicitly defined nor sufficiently supported by the data at the time it is introduced. "Molecular memory" generally implies a persistent molecular state that remains after the removal of the original stimulus. Initially, it is unclear how the authors distinguish this from a transient transcriptional response to drug exposure. Furthermore, the significance of Figure 7A as the primary evidence for this memory is under-explained; a Venn diagram of gene counts does not inherently prove a biological memory without careful biological interpretation.

Please define "molecular memory" early in the text and add references.

Please elaborate on the interpretation of Figure 7A in the main text. State clearly that the key evidence for memory is the stark contrast in the scale and nature of the response. This will effectively convince the reader that the Venn diagram provides quantitative evidence for a sophisticated, memory-driven state. Finally, clarify whether the same genes or pathways remain differentially expressed across the relevant comparisons to further strengthen the memory hypothesis.*

Response:

We agree that "molecular memory" requires a rigorous definition and clearer contextualization early in the manuscript to distinguish it from a transient transcriptional response. To address your comment, we have made the following revisions to the manuscript:

1. Defined "molecular memory" in the Introduction (Page 3-4, Lines 77-87 in unmarked clean version) as a sustained alterations in molecular states (e.g., epigenetic adaptations or epitranscriptomic RNA modifications) that persist after the initial stimulus is removed, citing foundational literature as shown below.
2. Expanded our discussion of Figure 7A in the Results section. We now explicitly highlight the stark contrast in the scale and nature of the response in drug-tolerant parasites, suggesting presence of memory-driven state, please see page 10, lines 412-416.
3. Regarding “clarify whether the same genes or pathways remain differentially expressed across the relevant comparisons to further strengthen the memory hypothesis”. It is extremely difficult bioinformatically to pinpoint what changes could be responsible for molecular memory in persisters across different comparisons and it is a goal of our further study. Therefore, to avoid overinterpretation we removed this sentence from Results section: “It is possible that due to the molecular memory in rechallenged persisters, many changes remain in place and therefore, driving to a coordinated and targeted response.” We have expanded the Discussion to elaborate how epigenetic and epitranscriptomic adaptations in other organisms might contribute to molecular memory, please see page 14, Lines 458-470. Additionally, we analyzed our data further and incorporated new information on potential epigenetic and epitranscriptomic modulators that shift in persisters and may drive this memory, please see lines 477-485 in unmarked clean version.

References added to the Introduction part:

1. Miyaue S, Suzuki E, Komiyama Y, Kondo Y, Morikawa M and Maeda S (2018) Bacterial Memory of Persisters: Bacterial Persister Cells Can Retain Their Phenotype for Days or Weeks After Withdrawal from Colony–Biofilm Culture. Front. Microbiol. 9:1396. doi: 10.3389/fmicb.2018.01396
2. Riber L, Hansen LH (2021) Epigenetic Memories: The Hidden Drivers of Bacterial Persistence? Trends in Microbiology 29 (3):190-194. doi:10.1016/j.tim.2020.12.005
3. Pashay Ahi E. Epitranscriptomics as a Candidate Universal Modulator of Dormancy Transitions. Ecol Evol. 2026 Feb 3;16(2):e73007. doi: 10.1002/ece3.73007. PMID: 41646876; PMCID: PMC12867678.
4. Shen, S., Faouzi, S., Bastide, A. *et al.* An epitranscriptomic mechanism underlies selective mRNA translation remodelling in melanoma persister cells. *Nat Commun* **10**, 5713 (2019). https://doi.org/10.1038/s41467-019-13360-6.

References added to the Discussion part:

1. Shen, S., Faouzi, S., Bastide, A. *et al.* An epitranscriptomic mechanism underlies selective mRNA translation remodelling in melanoma persister cells. *Nat Commun* **10**, 5713 (2019). https://doi.org/10.1038/s41467-019-13360-6
2. Zhao X, Guan S. Epitranscriptomic control of cancer immunity and therapy resistance. Front Immunol. 2025 Nov 7;16:1706557. doi: 10.3389/fimmu.2025.1706557. PMID: 41280938; PMCID: PMC12634331.
3. Riber L, Hansen LH. Epigenetic Memories: The Hidden Drivers of Bacterial Persistence? Trends Microbiol. 2021 Mar;29(3):190-194. doi: 10.1016/j.tim.2020.12.005. Epub 2021 Jan 4. PMID: 33414017.
4. Pashay Ahi E. Epitranscriptomics as a Candidate Universal Modulator of Dormancy Transitions. Ecol Evol. 2026 Feb 3;16(2):e73007. doi: 10.1002/ece3.73007. PMID: 41646876; PMCID: PMC12867678.

*4. Lack of Functional Validation
The study's mechanistic conclusions are drawn entirely from transcriptomic data. The authors frequently make functional claims (e.g., "remodeling of protein synthesis machinery") with the assumption that mRNA levels correlate directly with protein abundance and function.

Please temper the language throughout the manuscript to reflect that these findings represent transcriptomic blueprints rather than confirmed functional states. The manuscript would be significantly strengthened by explicitly acknowledging this limitation in the Discussion and suggesting future proteomic or functional assays (e.g., translation rate measurements) to validate these observations.*

Response:

We have tempered the language throughout the manuscript, acknowledge this as a limitation of the study in the Discussion (Study limitations and future directions section: pages 15-16, lines 554-568 in unmarked clean version) and proposed proteomics and polysome profiling experiments to validate our findings in the future.

Reviewer #3: No extra experiments are requested

**Part III – Minor Issues: Editorial and Data Presentation Modifications**
Please use this section for editorial suggestions as well as relatively minor modifications of existing data that would enhance clarity.

*Reviewer #1: (No Response)*

*Reviewer #2:*

Thank you for your valuable comments. Please see our point-by-point responses below.

*1. Limitations of the In Vitro Model.
The in vitro model is a strength for its tractability, but its limitations in representing the in vivo context are not adequately discussed. The authors should add a paragraph to the Discussion explicitly acknowledging the absence of host immune pressure (e.g., oxidative stress, nutrient deprivation) and the use of insect-stage promastigotes rather than clinically relevant amastigotes. It is highly probable that an in vivo persister transcriptome would reflect a composite response to both drug and host immunity.*

Response:

Yes, we agree that in vitro model has limitations compared to in vivo model. We have described the limitations of our in vitro model and proposed a development of in vivo amastigote model as our future goal in the Study Limitations and Future Directions section, pages 15-16, lines 554-568 in unmarked clean version.

*2. Single Time-Point Analysis
The transcriptomic analysis for each stage relies on a single time point. Because persistence is a dynamic process, capturing it at one snapshot (e.g., 24 hours post-drug) may miss crucial early stress responses or late stabilization events. Briefly note in the discussion that future time-course studies would be highly beneficial.*

Response

We have added Study Limitations and Future Directions section in the Discussion to include this sentence: “Furthermore, while this study evaluated drug responses at a single time point, future work will utilize time-course experiments to track early and late cellular changes in persister-like parasites.”

*3. The MTT assay results are presented as color changes on the assay plates; however, it is not immediately clear how the different colors should be interpreted. Although this is briefly described in the legend for Supplementary Figure 3, it would be helpful to include a concise explanation in the main results section (and/or Methods) so that readers can more easily understand how the color changes relate to cell viability and support the conclusions drawn from the assay.*

We have revised the manuscript to explain color changes in MTT assay in Results section (page 6, lines 162-164 in clean unmarked version).

*4. The manuscript would benefit from defining all acronyms at their first occurrence to improve readability. In addition, please provide appropriate references for established experimental methods (e.g., the MTT assay and other standard methodologies) in the Methods section where applicable.*

Response:

We have defined all acronyms at their first occurrence and added references for MTT assay and other standard methodologies in the Methods section.

5. The quality the micrographs including in Supplementary Figure 2 could be improved by providing higher-resolution images, as some details are difficult to appreciate at the current resolution. In addition, please indicate the magnification (or include scale bars where appropriate) for all microscopy images to facilitate interpretation and reproducibility.

Response:

**Please note that some cells appear out of focus due to the nature of liquid culture preparations.** Unfortunately, alternative imagery is unavailable. However, we have **included the specific magnification details** within the figure for clarity.

6. In the legend for Figure S3, it is stated that the data are presented as mean ± standard error; however, Figure S3 does not appear to contain any graphs. Please revise the legend as necessary.

Response:

Sorry, it is our mistake. The legend has been revised accordingly.

*Reviewer #3: • In the abstract, Authors mention that persistence is defined as “drug-susceptible” parasites, however their model of persistence consists of selecting drug-resistant parasites by exposing them to antimonial drugs. It is contradictory to then propose this model as a persistence model. I recommend revising the definition of persistence.*

We would like to thank the reviewer for valuable comments.

*• The definition of persistence is not a problem in the introduction section, only on how its defined in the abstract*

Response:

We revised definition of persistence in abstract. Our sentence has been modified like this: “Persistence - characterized by the transient ability of subpopulations of *Leishmania* parasites to survive exposure to drug - is a major driver of treatment failure and clinical relapses in leishmaniasis.” (page 2, Lines 11-13 in unmarked clean version).

*• In line 12 PAT should be defined (pentavalent antimonial or potassium antimonyl
tartrate??)*

Response:

We defined the drug name (potassium antimonyl tartrate) in the Abstract, please see line 17 in unmarked clean version.

*• When generating persister-like leishmania, authors selected to use EC80 of PAT in their parasite cultures. However, it is not clear why they selected this and not any other concentration. If there is literature reporting that 80% of the parasites are killed during drug treatment, this should be stated. Otherwise, the election of this number should be backed up.*

Response:

We choose EC80 to kill 80% of parasites because this condition provided sufficient cell numbers for downstream experiments, including deep RNA-seq and examination of drug tolerance. We modified sentence in the results section (lines 138-140 in unmarked clean version) to clarify that: “This concentration, which reduces the live parasite population to 20% (persister-like), was selected for all follow-up experiments because it yields enough cells for deep RNA-seq and drug tolerance studies.”

*• In line 244 “comparisons” has a typo*

Response:

Fixed.

*• Authors selected correctly their groups and the comparisons among them, and this selection process is very well explained. However, I consider that a graphical model of biological processes enriched and decreased would make the paper easier to follow and would help the reader understand the final message of the paper. For that reason, I recommend to create a figure/diagram that includes the information described in lines 277-299, table 1 and discussion section.*

We have prepared figure 8 that represents a graphical model of biological processes observed. It was added into Discussion section.

*• Authors affirm in line 270 that “This downregulation [of protein synthesis machinery] correlated with the increase in the drug tolerance observed upon rechallenge (Fig. 2).” Nevertheless, no correlation analysis was performed.*

Response:

We have revised the text to replace "correlated" with "coincided" to accurately state that these two events occurred at the same time.

New sentence: This downregulation [of protein synthesis machinery] coincided with the increase in the drug tolerance observed upon rechallenge (Fig. 2).” Please see line 296-297.

*• Why did authors select to use potassium antimonyl tartrate rather than a drug treatment that is commonly used in the clinic?*

Response:

We chose potassium antimonyl tartrate (PAT) because it represents the active form of the drug (SbIII) and is regularly used in ***in vitro* models (promastigotes and amastigotes)** as a reference standard and benchmark tool to study drug susceptibility, mechanism of action, and resistance pathways in Leishmania parasites.
