# Supplementary material for: Active transcriptome remodeling underlies antimony-induced drug tolerance in persister-like Leishmania parasites
Source: PLoS Pathog. 2026 Oct 5;22(10):e1014682. doi: 10.1371/journal.ppat.1014682 (PMC13649148; doi:10.1371/journal.ppat.1014682)
Supplement: Peer review history

# Peer review: Decision Letter 0

**DOI:** 10.1371/journal.ppat.1014682.r001

**Authors:** Tracey J. Lamb, Hira L. Nakhasi

17 Jul 2026

PPATHOGENS-D-26-01390

Active transcriptome remodeling and epigenetic adaptations underlie persistence in Leishmania parasites

PLOS Pathogens

Dear Dr. Karamysheva,

Thank you for submitting your manuscript to PLOS Pathogens. After careful consideration, we feel that it has merit but does not fully meet PLOS Pathogens's publication criteria as it currently stands. Therefore, we invite you to submit a revised version of the manuscript that addresses the points raised during the review process.

Please submit your revised manuscript by Sep 15 2026 11:59PM. If you will need more time than this to complete your revisions, please reply to this message or contact the journal office at plospathogens@plos.org. When you're ready to submit your revision, log on to https://www.editorialmanager.com/ppathogens/ and select the 'Submissions Needing Revision' folder to locate your manuscript file.

Please include the following items when submitting your revised manuscript:

* A letter that responds to each point raised by the editor and reviewer(s). You should upload this letter as a separate file labeled 'Response to Reviewers'. This file does not need to include responses to any formatting updates and technical items listed in the 'Journal Requirements' section below.

* A marked-up copy of your manuscript that highlights changes made to the original version. You should upload this as a separate file labeled 'Revised Manuscript with Track Changes'.

* An unmarked version of your revised paper without tracked changes. You should upload this as a separate file labeled 'Manuscript'.

If you would like to make changes to your financial disclosure, competing interests statement, or data availability statement, please make these updates within the submission form at the time of resubmission. Guidelines for resubmitting your figure files are available below the reviewer comments at the end of this letter. As the corresponding author, your ORCID iD is verified in the submission system and will appear in the published article. PLOS supports the use of ORCID, and we encourage all coauthors to register for an ORCID iD and use it as well. Please encourage your coauthors to verify their ORCID iD within the submission system before final acceptance, as unverified ORCID iDs will not appear in the published article. Only the individual author can complete the verification step; PLOS staff cannot verify ORCID iDs on behalf of authors.

We look forward to receiving your revised manuscript.

Kind regards,

Hira L Nakhasi, Ph.D.

Academic Editor

PLOS Pathogens

Tracey Lamb

Section Editor

PLOS PathogensSumita Bhaduri-McIntosh

Editor-in-Chief

PLOS Pathogens

orcid.org/0000-0003-2946-9497

Michael Malim

Editor-in-Chief

PLOS Pathogens

orcid.org/0000-0002-7699-2064

**Additional Editor Comments (if provided):**

**Journal Requirements:**

If the reviewer comments include a recommendation to cite specific previously published works, please review and evaluate these publications to determine whether they are relevant and should be cited. There is no requirement to cite these works unless the editor has indicated otherwise.

1) We ask that a manuscript source file is provided at Revision. Please upload your manuscript file as a .doc, .docx, .rtf or .tex. If you are providing a .tex file, please upload it under the item type u2018LaTeX Source Fileu2019 and leave your .pdf version as the item type u2018Manuscriptu2019.

2) We do not publish any copyright or trademark symbols that usually accompany proprietary names, eg ©,  ®, or TM  (e.g. next to drug or reagent names). Therefore please remove all instances of trademark/copyright symbols throughout the text, including:

- ® on pages: 16, and 17

- TM on page: 16.

3) Please upload all main figures as separate Figure files in .tif or .eps format. For more information about how to convert and format your figure files please see our guidelines:

https://journals.plos.org/plospathogens/s/figures

4) Please upload a copy of Figure Fig 1-7 which you refer to in your text on pages 5, 6, 7, and 9. Or, if the figure is no longer to be included as part of the submission please remove all reference to it within the text.

5) We notice that your supplementary Figures are included in the manuscript file. Please remove them and upload them with the file type 'Supporting Information'. Please ensure that each Supporting Information file has a legend listed in the manuscript after the references list.

6) Please amend your detailed Financial Disclosure statement. This is published with the article. It must therefore be completed in full sentences and contain the exact wording you wish to be published.

i) State what role the funders took in the study. If the funders had no role in your study, please state: "The funders had no role in study design, data collection and analysis, decision to publish, or preparation of the manuscript."

ii) If any authors received a salary from any of your funders, please state which authors and which funders..

If you did not receive any funding for this study, please simply state: u201cThe authors received no specific funding for this work.u201d

7) We have amended your Competing Interest statement to comply with journal style. We kindly ask that you double check the statement and let us know if anything is incorrect.

**Reviewers' Comments:**

Reviewer's Responses to Questions

**Part I - Summary**

Please use this section to discuss strengths/weaknesses of study, novelty/significance, general execution and scholarship.

Reviewer #1: The manuscript by Quienco et al., describes an experimental system using an in vitro drug exposure/recovery/rechallenge model in promastigotes. The terminology used throughout the manuscript is problematic. The authors refer to drug-surviving axenic promastigotes as “persisters” and repeatedly link this model to persistence and clinical relapse. However, persistent Leishmania infection is generally studied in the context of host infection, particularly intracellular amastigotes within mammalian cells or tissues, rather than axenic promastigote cultures. The phenotype observed in vitro in their drug-exposure/recovery/rechallenge model i.e., survival after EC80 antimony exposure, delayed regrowth, and increased EC50 upon rechallenge, is more accurately described as drug tolerance, stress adaptation, or drug-tolerant promastigote survival. Unless the authors demonstrate reversibility, lack of heritable resistance, absence of genomic amplification/mutation, and relevance in intracellular amastigotes or an infection model, the term “persister” should be used with substantial caution, preferably as “persister-like,” and the title, abstract, and conclusions should be revised to avoid conflating this phenotype with persistent Leishmania infection.

1) The term "persistence" as commonly understood in the context of infectious disease carries specific connotations that the axenic promastigote model does not fully satisfy. In in vivo settings, persistence occurs within phagocytic cells (macrophages), where parasites must overcome host immune mechanisms including reactive oxygen and nitrogen species, acidic pH, nutrient limitation, and active immune surveillance. None of these pressures are recapitulated in axenic promastigote culture employed in this study. The drug pressure applied here is a single, defined chemical stressor, a significant simplification of the hostile host environment.

2) Amastigote persistence within macrophages involves complex host-parasite interactions, including manipulation of host cell signaling, evasion of phagolysosomal killing, and modulation of antigen presentation. Promastigotes in axenic culture exist in none of this biological context.

3) The promastigote parasites are adapted to the sandfly midgut environment, not the mammalian host. Their baseline biology including their metabolic preferences, surface molecule expression, and stress response repertoire differs substantially from amastigotes. Drug responses observed in promastigotes may not translate directly to amastigote behavior in vivo.

4) The EC80 PAT challenge used in this study selects for cells that can survive a specific chemical insult. Whether the surviving cells are genuinely "persisters" in the biological sense, or simply the tail of a normal distribution of drug sensitivity within the population, is not definitively resolved by the data presented. The authors do not perform single-cell analyses or lineage tracing to confirm that the surviving cells are a pre-existing subpopulation rather than stochastically drug-tolerant cells.

5) Leishmania parasites use polycistronic transcription, which is an important context here. Trypanosomatids largely lack conventional single-gene transcriptional regulation; genes are transcribed in polycistronic transcription units and mature mRNAs are generated by trans-splicing and polyadenylation, with much regulation occurring post-transcriptionally. The claims about 'active transcriptome remodeling' is not impossible, but it needs to be interpreted carefully. RNA abundance changes in Leishmania can reflect mRNA stability, processing, copy-number variation, translation-linked RNA turnover, aneuploidy, or selection of subpopulations, not necessarily classical transcriptional activation/repression.

6) Following on the comment above, the claims regarding “epigenetic adaptations” are not sufficiently supported by the presented data. Leishmania does possess chromatin-associated regulatory mechanisms, including histone modifications and base J, but gene expression in this parasite is organized primarily through polycistronic transcription units and extensive post-transcriptional regulation. Therefore, changes in steady-state mRNA abundance cannot be straightforwardly interpreted as evidence of epigenetic regulation. The manuscript appears to infer epigenetic remodeling mainly from differential expression of putative histone-modifying enzymes. This is insufficient to support the claim made in the title of the manuscript that epigenetic adaptations underlie the phenotype. Direct evidence, such as ChIP-seq/ChIP-qPCR for relevant histone marks, base J mapping, ATAC-seq/MNase-seq, or perturbation of the proposed epigenetic regulators, would be required. In the absence of such data, the authors should substantially tone down the epigenetic claims and describe them as hypotheses generated from transcriptomic data. The authors are clearly overinterpreting the data.

7) Overall, the manuscript is presented as a study of Leishmania persistence, but the data primarily support an in vitro model of antimony-induced drug tolerance/adaptation in axenic promastigotes. The title, abstract, and discussion overstate the connection to persistent infection and clinical relapse. The terminology is especially confusing because persistent Leishmania infection is usually associated with intracellular amastigote stages in host cells or tissues, whereas this study uses promastigote cultures. In addition, the claims of “epigenetic adaptations” are not directly demonstrated. Given the polycistronic organization of Leishmania gene expression and the dominant role of post-transcriptional regulation, RNA-seq changes alone do not establish epigenetic remodeling. The manuscript would be stronger and more accurate if reframed as a study of drug-tolerant promastigote survival and transcriptomic/post-transcriptional adaptation following antimony exposure, with epigenetic mechanisms discussed only as a speculative possibility requiring direct validation.

Reviewer #2: This manuscript presents a well-designed and comprehensive study addressing a significant gap in our understanding of treatment failure in leishmaniasis. The authors establish an in vitro model using Ficoll gradient centrifugation to isolate persister-like Leishmania mexicana parasites and utilize RNA-seq to investigate the molecular mechanisms of persistence. A major strength of the study is the multi-stage experimental design (induction, recovery, and re-challenge), which reveals that persistence is an active, orchestrated process rather than a passive metabolic shutdown. Furthermore, the identification of a "transcriptomic memory" in re-challenged persisters is a highly valuable contribution to the field. While the data is robust and the overall conclusions are sound, the manuscript would benefit from adjustments to its narrative, improved data visualization, and expanded discussion of the model's limitations

Reviewer #3: In the manuscript entitled “Active transcriptome remodeling and epigenetic adaptations underlie persistence in Leishmania parasites” Quiceno and colleagues perform transcriptome analysis in a model of parasite persistence. They have demonstrated the biological processes and pathways involved in the generating of persistence after drug treatment and the changes once the treatment is removed. I consider this paper to be relevant for improving drug treatments against leishmaniasis. Still, some questions and comments should be addressed.

**********

**Part II – Major Issues: Key Experiments Required for Acceptance**

Please use this section to detail the key new experiments or modifications of existing experiments that should be absolutely required to validate study conclusions.

Generally, there should be no more than 3 such required experiments or major modifications for a "Major Revision" recommendation. If more than 3 experiments are necessary to validate the study conclusions, then you are encouraged to recommend "Reject".

Reviewer #1: 1) The metabolic restriction in amastigotes reported in studies of parasite persistence by Beverley et al., and McConville et al., report it as a developmental program, not a stress response. The slow doubling time and restricted metabolism of amastigotes reported by Beverley et al., reflect a genetically and epigenetically encoded developmental differentiation, not a transient phenotypic adaptation to drug pressure. Conflating the two risks overstating the novelty of the persister phenotype described in this study, since some of what is being observed in drug-stressed promastigotes may simply reflect partial activation of the amastigote developmental program under stress — a well-known phenomenon in Leishmania biology.

Reviewer #2: 1. Presentation and Visualization of Transcriptomic Data

While the text provides data supporting the claim of "extensive transcriptome remodeling," the current presentation relies heavily on dense textual descriptions of gene counts. This makes it difficult for the reader to understand the scale, direction, and dynamics of these drastic changes.

Please add a consolidated table or a stacked bar chart clearly summarizing the absolute number of up-regulated versus down-regulated genes across all comparisons side-by-side. Consolidate the Results text.

2. Premature Conclusion of a "Dormant State"

The conclusion that persister parasites enter a "dormant state" is introduced prematurely in the first Results section. The primary evidence presented at that stage is a population-level growth curve (Fig. 2A) demonstrating delayed proliferation. While consistent with dormancy, this correlational phenotype could equally stem from a generalized recovery from cellular damage.

Please present the findings strictly as phenotypes (delayed proliferation and enhanced drug tolerance). Reserve the mechanistic claim of a "dormant state" until after presenting the transcriptomic data, which provides the necessary molecular evidence (e.g., global downregulation of biosynthetic processes) to support regulated quiescence.

3. Clarifying the Concept and Evidence for "Molecular Memory"

The authors repeatedly refer to "molecular memory," yet this central concept is neither explicitly defined nor sufficiently supported by the data at the time it is introduced. "Molecular memory" generally implies a persistent molecular state that remains after the removal of the original stimulus. Initially, it is unclear how the authors distinguish this from a transient transcriptional response to drug exposure. Furthermore, the significance of Figure 7A as the primary evidence for this memory is under-explained; a Venn diagram of gene counts does not inherently prove a biological memory without careful biological interpretation.

Please define "molecular memory" early in the text and add references.

Please elaborate on the interpretation of Figure 7A in the main text. State clearly that the key evidence for memory is the stark contrast in the scale and nature of the response. This will effectively convince the reader that the Venn diagram provides quantitative evidence for a sophisticated, memory-driven state. Finally, clarify whether the same genes or pathways remain differentially expressed across the relevant comparisons to further strengthen the memory hypothesis.

4. Lack of Functional Validation

The study's mechanistic conclusions are drawn entirely from transcriptomic data. The authors frequently make functional claims (e.g., "remodeling of protein synthesis machinery") with the assumption that mRNA levels correlate directly with protein abundance and function.

Please temper the language throughout the manuscript to reflect that these findings represent transcriptomic blueprints rather than confirmed functional states. The manuscript would be significantly strengthened by explicitly acknowledging this limitation in the Discussion and suggesting future proteomic or functional assays (e.g., translation rate measurements) to validate these observations.

Reviewer #3: No extra experiments are requested

**********

**Part III – Minor Issues: Editorial and Data Presentation Modifications**

Please use this section for editorial suggestions as well as relatively minor modifications of existing data that would enhance clarity.

Reviewer #1: (No Response)

Reviewer #2: 1. Limitations of the In Vitro Model.

The in vitro model is a strength for its tractability, but its limitations in representing the in vivo context are not adequately discussed. The authors should add a paragraph to the Discussion explicitly acknowledging the absence of host immune pressure (e.g., oxidative stress, nutrient deprivation) and the use of insect-stage promastigotes rather than clinically relevant amastigotes. It is highly probable that an in vivo persister transcriptome would reflect a composite response to both drug and host immunity.

2. Single Time-Point Analysis

The transcriptomic analysis for each stage relies on a single time point. Because persistence is a dynamic process, capturing it at one snapshot (e.g., 24 hours post-drug) may miss crucial early stress responses or late stabilization events. Briefly note in the discussion that future time-course studies would be highly beneficial.

3. The MTT assay results are presented as color changes on the assay plates; however, it is not immediately clear how the different colors should be interpreted. Although this is briefly described in the legend for Supplementary Figure 3, it would be helpful to include a concise explanation in the main results section (and/or Methods) so that readers can more easily understand how the color changes relate to cell viability and support the conclusions drawn from the assay.

4. The manuscript would benefit from defining all acronyms at their first occurrence to improve readability. In addition, please provide appropriate references for established experimental methods (e.g., the MTT assay and other standard methodologies) in the Methods section where applicable.

5. The quality the micrographs including in Supplementary Figure 2 could be improved by providing higher-resolution images, as some details are difficult to appreciate at the current resolution. In addition, please indicate the magnification (or include scale bars where appropriate) for all microscopy images to facilitate interpretation and reproducibility.

6. In the legend for Figure S3, it is stated that the data are presented as mean ± standard error; however, Figure S3 does not appear to contain any graphs. Please revise the legend as necessary.

Reviewer #3: • In the abstract, Authors mention that persistence is defined as “drug-susceptible” parasites, however their model of persistence consists of selecting drug-resistant parasites by exposing them to antimonial drugs. It is contradictory to then propose this model as a persistence model. I recommend revising the definition of persistence.

• The definition of persistence is not a problem in the introduction section, only on how its defined in the bstract

• In line 12 PAT should be defined (pentavalent antimonial or potassium antimonyl

tartrate??)

• When generating persister-like leishmania, authors selected to use EC80 of PAT in their parasite cultures. However, it is not clear why they selected this and not any other concentration. If there is literature reporting that 80% of the parasites are killed during drug treatment, this should be stated. Otherwise, the election of this number should be backed up.

• In line 244 “comparisons” has a typo

• Authors selected correctly their groups and the comparisons among them, and this selection process is very well explained. However, I consider that a graphical model of biological processes enriched and decreased would make the paper easier to follow and would help the reader understand the final message of the paper. For that reason, I recommend to create a figure/diagram that includes the information described in lines 277-299, table 1 and discussion section.

• Authors affirm in line 270 that “This downregulation [of protein synthesis machinery] correlated with the increase in the drug tolerance observed upon rechallenge (Fig. 2).” Nevertheless, no correlation analysis was performed.

• Why did authors select to use potassium antimonyl tartrate rather than a drug treatment that is commonly used in the clinic?

**********

PLOS authors have the option to publish the peer review history of their article (what does this mean?). If published, this will include your full peer review and any attached files.

If you choose “no”, your identity will remain anonymous but your review may still be made public.

**Do you want your identity to be public for this peer review?**  For information about this choice, including consent withdrawal, please see our Privacy Policy.

Reviewer #1: **Yes:**  Sreenivas Gannavaram

Reviewer #2: No

Reviewer #3: No

[NOTE: If reviewer comments were submitted as an attachment file, they will be attached to this email and accessible via the submission site. Please log into your account, locate the manuscript record, and check for the action link "View Attachments". If this link does not appear, there are no attachment files.]

**Figure resubmission:**

While revising your submission, we strongly recommend that you use PLOS’s NAAS tool (https://ngplosjournals.pagemajik.ai/artanalysis) to test your figure files. NAAS can convert your figure files to the TIFF file type and meet basic requirements (such as print size, resolution), or provide you with a report on issues that do not meet our requirements and that NAAS cannot fix.

After uploading your figures to PLOS’s NAAS tool - https://ngplosjournals.pagemajik.ai/artanalysis, NAAS will process the files provided and display the results in the "Uploaded Files" section of the page as the processing is complete. If the uploaded figures meet our requirements (or NAAS is able to fix the files to meet our requirements), the figure will be marked as "fixed" above. If NAAS is unable to fix the files, a red "failed" label will appear above. When NAAS has confirmed that the figure files meet our requirements, please download the file via the download option, and include these NAAS processed figure files when submitting your revised manuscript.

**Reproducibility:**

To enhance the reproducibility of your results, we recommend that authors of applicable studies deposit laboratory protocols in protocols.io, where a protocol can be assigned its own identifier (DOI) such that it can be cited independently in the future. Additionally, PLOS ONE offers an option to publish peer-reviewed clinical study protocols. Read more information on sharing protocols at https://plos.org/protocols?utm_medium=editorial-email&utm_source=authorletters&utm_campaign=protocols

# Author comment: Author response to Decision Letter 1

**DOI:** 10.1371/journal.ppat.1014682.r002

17 Sep 2026

Attachment Submitted filename: Response-to-Reviewers-PPATHOGENS-AddressingComments.docx

# Editor report: Decision Letter 1

**DOI:** 10.1371/journal.ppat.1014682.r003

**Authors:** Tracey J. Lamb, Hira L. Nakhasi

22 Sep 2026

Dear Karamysheva,

We are pleased to inform that your manuscript, "Active transcriptome remodeling underlies antimony-induced drug tolerance in persister-like Leishmania parasites", has been editorially accepted for publication at PLOS Pathogens.

Before your manuscript can be formally accepted and sent to production, you will need to complete our formatting changes, which you will receive by email within a week. Please note that your manuscript will not be scheduled for publication until you have made the required changes.

IMPORTANT NOTES

(1) Copyediting and Proofreading: The corresponding author will receive a typeset proof for review, to ensure errors have not been introduced during production. Please review the PDF proof of your manuscript carefully, as this is the last chance to correct any errors. Please note that major changes, or those which affect the scientific understanding of the work, will likely cause delays to the publication date of your manuscript.

(2) Appropriate Figure Files: Please remove all name and figure # text from your figure files. Please also take this time to check that your figures are of high resolution, which will improve the readbility of your figures and help expedite your manuscript's publication. Please note that figures must have been originally created at 300dpi or higher. Do not manually increase the resolution of your files. For instructions on how to properly obtain high quality images, please review our Figure Guidelines, with examples at: http://journals.plos.org/plospathogens/s/figures.

We encourage you to use PACE (http://pace.apexcovantage.com/), a figure-checking tool which helps authors ensure all figures meet PLOS requirements so that figures can publish in the highest quality. PACE is a digital diagnostic and conversion tool for figure files. It will provide information about any failed check(s) and, if able, will automatically convert the figure into an acceptable file that passes quality checks.

To use PACE, you must first register as a user. Then, login and navigate to the UPLOAD tab, where you will find detailed instructions on how to use the tool.

(3) Striking Image: Please upload a striking still image to accompany your article if one is available (you can include a new image or an existing one from within your manuscript). Should your paper be accepted, this image will be considered for our monthly issue image and may also appear on our website to feature your article. Please upload this as a separate file, selecting "striking image" as the file type upon upload. Please also include a separate "Other" file with a caption, including credits and any potential copyright information. Please do not include the caption in the main article file. If your image is from someone other than yourself, please ensure that the artist has read and agreed to the terms and conditions of the Creative Commons Attribution License at http://journals.plos.org/plospathogens/s/content-license. Please note that PLOS cannot publish copyrighted images.

(4) Press Release or Related Media: If you or your institution will be preparing press materials for this manuscript, please inform our press team in advance at plospathogens@plos.org as soon as possible. We ask that you contact us within one week to plan ahead of our fast Production schedule. If you need to know your paper's publication date for related media purposes, you must coordinate with our press team, and your manuscript will remain under a strict press embargo until the publication date and time.

(5) Update your Profile Information: Now that your manuscript has been provisionally accepted, please log into Editorial Manager and update your profile, if needed. Go to http://www.editorialmanager.com/ppathogens, log in, and click on the "Update My Information" link at the top of the page. Please update your user information to ensure an efficient production and billing process.

(6) LaTeX users only: Our staff will ask you to upload a TEX file in addition to the PDF before the paper can be sent to typesetting, so please carefully review our Latex Guidelines http://journals.plos.org/plospathogens/s/latex in the meantime.

Thank you again for supporting Open Access publishing; we are looking forward to publishing your work in PLOS Pathogens.

Best regards,

Hira L Nakhasi, Ph.D.

Academic Editor

PLOS Pathogens

Tracey Lamb

Section Editor

PLOS Pathogens

Dominique Soldati-Favre

Editor-in-Chief

PLOS Pathogens

***********************************************************

# Editor report: Acceptance letter

**DOI:** 10.1371/journal.ppat.1014682.r004

**Authors:** Tracey J. Lamb, Hira L. Nakhasi

Dear Associate Professor Karamysheva,

We are delighted to inform you that your manuscript, "Active transcriptome remodeling underlies antimony-induced drug tolerance in persister-like Leishmania parasites," has been formally accepted for publication in PLOS Pathogens.

We have now passed your article onto the PLOS Production Department who will complete the rest of the pre-publication process. All authors will receive a confirmation email upon publication.

Thank you again for supporting open-access publishing; we are looking forward to publishing your work in PLOS Pathogens.

Best regards,

Dominique Soldati-Favre

Editor-in-Chief

PLOS Pathogens
